# Elemental Profiling of Single Bacterial Cells As a Function of Copper Exposure and Growth Phase

**DOI:** 10.1371/journal.pone.0021255

**Published:** 2011-06-16

**Authors:** Ran Yu, Barry Lai, Stefan Vogt, Kartik Chandran

**Affiliations:** 1 Department of Earth and Environmental Engineering, Columbia University, New York, New York, United States of America; 2 Advanced Photon Source, Argonne National Laboratory, Argonne, Illinois, United States of America; Auburn University, United States of America

## Abstract

The elemental composition of single cells of *Nitrosomonas europaea* 19718 was studied via synchrotron X-ray fluorescence microscopy (XFM) as a function of inhibition by divalent copper (Cu(II)) and batch growth phase. Based on XFM, the intracellular Cu concentrations in exponential phase cultures of *N. europaea* exposed to Cu(II) were statistically higher than in stationary phase cultures at the 95% confidence interval (α = 0.05). However, the impact of Cu inferred from specific oxygen uptake rate (sOUR) measurements at the two physiological states was statistically not dissimilar at the Cu(II) doses tested, except at 1000 µM Cu(II), at which exponential phase cultures were significantly more inhibited. Furthermore, the elemental composition in uninhibited exponential and stationary phase *N. europaea* cultures was similar. Notably, the molar fractions of Cu and Fe, relative to other elements in *N. europaea* cultures were statistically higher than those recently reported in *Pseudomonas fluorescens* possibly owing to the preponderance of metal cofactor rich catalytic enzymes (such as ammonia monooxygenase) and electron transport mechanisms in *N. europaea*.

## Introduction

Metals are widely prevalent in nature and as such interact extensively with all life forms. On one hand, metals are recalcitrant environmental pollutants, introduced into the environment by several industrial activities. On the other hand, metals such as iron, copper, manganese, zinc, nickel and molybdenum are widely involved in bacterial metallo-enzymes [1] and catalyze a wide array of biochemical reactions including those involved in essential metabolism and stress resistance. Traditionally, the metal composition of bacterial cells has been approximated and measured using bulk elemental analysis [1]. However, there are few reports pertaining to the *in-situ* inspection and quantification of the metal concentrations in bacterial cells. For instance, although there exists some knowledge of metal ‘quotas’ in model bacteria such as *Escherichia coli* [2], [3], little is known if and how bacteria modulate their elemental composition in response to factors such as substrate sufficiency, starvation or toxicant stress.

From a toxicity perspective, it could also be useful to correlate intracellular concentrations of toxicants, such as heavy metals with whole-cell toxic responses. Indeed, it has been previously shown that intracellular metal concentrations and metal speciation ultimately govern metal toxicity and correlate well with bacterial toxic responses rather than total metal concentrations or dosage in the bulk-liquid phase [4].

**Figure 1:**
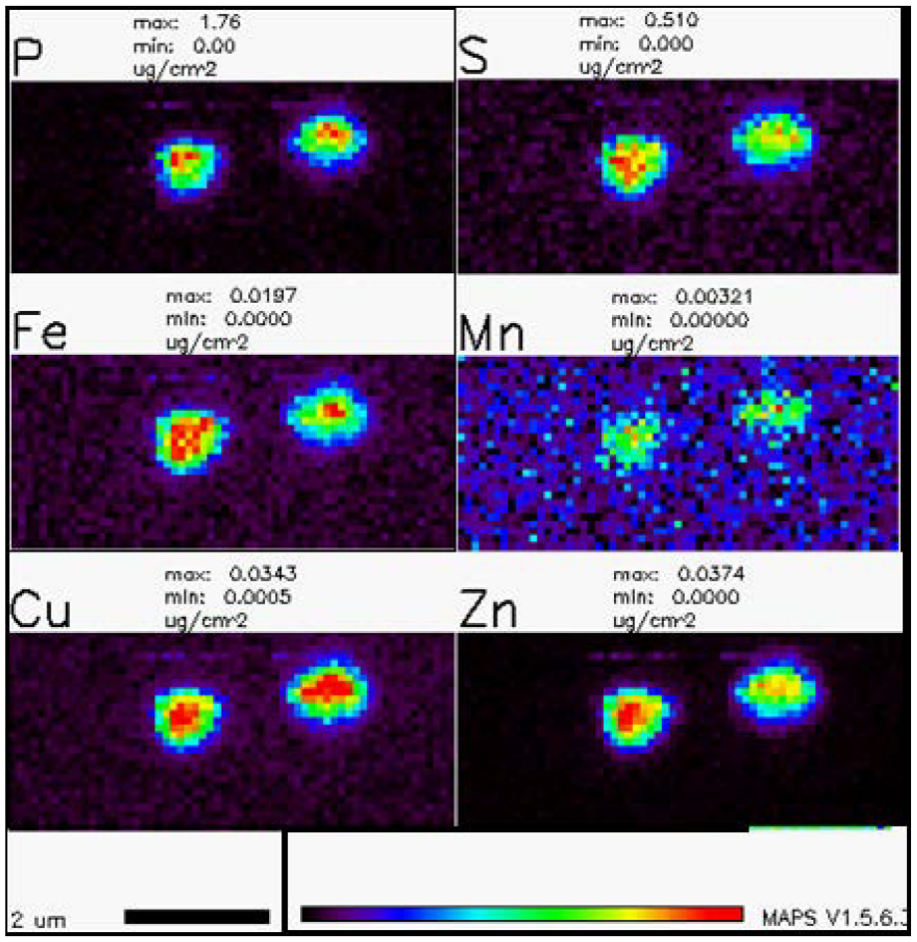
Spatial profiles of several elements in two *N. europaea* cells in close proximity at stationary phase and not exposed to Cu(II), quantified using MAPS software [**33**]. Dark colors represent lower concentrations and lighter colors represent higher concentrations.

Recently, synchrotron XFM has emerged as a viable tool for the noninvasive characterization of hydrated cells with a spatial resolution of about 150 nm [5]. Synchrotron XFM is especially appealing since it permits both spatial mapping and determination of concentrations and oxidation states of intracellular elements, without the need for cell lysis and extraction, for instance, as presented in Figure 1 [5]. Therefore, the overall goal of this study was to employ synchrotron XFM to determine changes in the elemental composition of *Nitrosomonas europaea* 19718, as a result of exposure to Cu(II) stress and as a function of physiological batch growth state. Copper is a widespread environmental pollutant and is speculated to be a cofactor of ammonia monooxygenase (AMO) [6], [7], [8] and nitrite reductase (NirK) [9] in *N. europaea*. Since actively growing cells of *N. europaea* are more susceptible to metal toxicity than stationary phase cultures [10], it was hypothesized that the higher toxicity observed during exponential phase would correspond with higher intracellular Cu concentrations for the same Cu(II) dose. Additionally, given the potential for copper and iron to play a primary role in *N. europaea* metabolism [6], [7], [8] it was hypothesized that *N. europaea* cells would be preferentially ‘enriched’ in these two elements compared to other bacteria. The specific objectives of this study were to: (1) examine the impact of physiological state (exponential and stationary phases during batch growth) on intracellular elemental composition as inferred from synchrotron XFM and (2) determine the impact of Cu(II) exposure at these physiological states on intracellular elemental composition and ammonia oxidation rates of *N. europaea*.

## Results and Discussion

### Impact of Cu(II) exposure on elemental composition

In keeping with our first hypothesis, exponentially growing *N. europaea* cultures exposed to copper had statistically higher intracellular Cu concentrations (α = 0.05) relative to stationary phase cultures (Tables 1 and 2). Additionally, there was an increasing trend in intracellular concentrations of P and S in exponential phase cultures exposed to Cu(II) relative to the control, for Cu(II) doses of 5 µM and 10 µM (Table 1). Specifically, for these Cu(II) doses, intracellular P and S concentrations were statistically higher in the exponential phase cultures than in the stationary phase cultures (Tables 1 and 2). At the higher Cu(II) doses of 100 µM and 1000 µM, the intracellular concentrations of P, S and Fe plateaued or declined for exponential phase cultures (Table 1). At these doses, the toxicity of Cu(II) possibly hindered the metabolic processes oriented towards sequestration and assimilation of these essential elements. In contrast, the concentrations of Zn in exponential phase cells (Table 1) and all non-Cu elements in stationary phase cells (Table 2) were largely non-systematically varying with Cu(II) exposure.

**Table 1:** Elemental profiles in exponential phase *N. europaea* cultures exposed to different Cu(II) doses.

Element	95% confidence intervals on elemental concentrations (µM) expressed to two significant figures				
	Control	5 µM	10 µM	100 µM	1000 µM*
**P**	77000–110000	100000–140000	130000–160000	150000–210000	110000
**S**	32000–43000	53000–72000	60000–70000	46000–69000	12000
**Fe**	3500–5000	3700–6900	7100–13000	1100–1700	960
**Cu**	1900–2600	3200–4900	5000–6200	62000–120000	150000
**Zn**	710–1100	610–1000	1200–1900	680–1300	3800
**n**	6	4	5	3	1

*: done without replication.

**Table 2:** Elemental profiles in stationary phase *N. europaea* cultures exposed to different Cu(II) doses.

Element	95% confidence intervals on elemental concentrations (µM) expressed to two significant figures				
	Control	5 µM	10 µM	100 µM	1000 µM
**P**	98000–110000	80000–90000	71000–86000	37000–82000	74000–84000
**S**	39000–45000	44000–48000	39000–43000	43000–62000	38000–43000
**Fe**	4100–4600	1800–2000	1800–2700	2300–3800	1300–1500
**Cu**	1700–2100	1900–2200	3200–4300	19000–49000	42000–53000
**Zn**	510–620	310–380	270–350	910–2400	360–430
**n**	6	5	5	7	5

The increased intracellular Cu concentrations in Cu(II) dosed cultures during exponential phase is to be expected given ATP driven Cu transport in *N. europaea* [9] with the higher energy available during exponential growth presumably facilitating increased Cu uptake in the Cu dosed cultures. Additionally, the markedly different elemental profiles in exponential phase and stationary phase *N. europaea* cultures exposed to Cu(II) points to different strategies employed to mitigate Cu(II) associated toxicity.

The higher uptake of P and S by exponential phase cultures into the cytoplasm at Cu(II) doses of 5 µM and 10 µM might be a means to sequester divalent copper cations (Cu^2+^) and render them unavailable to bind with biologically active molecules and moieties such as the sulfhydryl groups of proteins. Furthermore, it has been shown even in early studies [11] that *N. europaea* has extensive intracellular membrane invaginations [11], which likely serve as an added line of defense to potential toxins, sites for membrane-bound proteins involved in substrate transport or energy synthesis or sites for proton translocation and generation of the proton motive force. Considering that prokaryotic membranes in general are rich in phospholipids, the higher concentrations of P in exponential phase cells of *N. europaea* could be associated with added synthesis of these secondary membrane structures. Indeed, a high phospholipid content has been measured in bacteria that contain such internal membranes including *N. europaea* in an early primary study [11]. The increased S concentrations may also be due to increased biosynthesis of glutathione to combat Cu(II) induced oxidative stress [12]. Increased synthesis of sulfur containing sacrificial targets of oxidative stressors is likely since *N. europaea* inherently lacks the glutathionie oxidoreductase gene [9] and cannot cycle between oxidized and reduced forms of glutathione. Presumably, this is one possible reason due to which exponential phase cells of *N. europaea* displayed higher intracellular S concentrations, as observed in this study (Table 1). On the other hand, the high energetic demand for synthesizing reduced sulfur compounds may not be feasible during stationary phase, which was possibly reflected in alternate Cu homeostasis mechanisms including decreased ATP dependent Cu uptake (Table 2).

### Impact of Cu(II) exposure on physiological and gene expression responses

**Figure 2:**
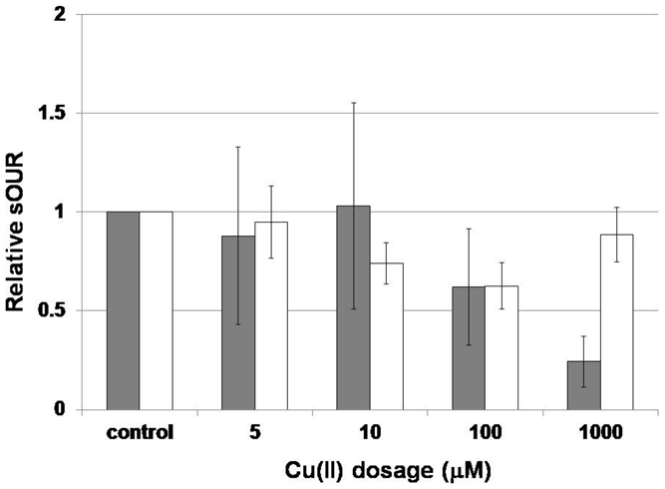
Impact of Cu(II) exposure on relative sOUR values, normalized to control values in stationary and exponential phase *N. europaea* cultures. Error bars depict standard deviation based on duplicate oxygen uptake rate measurements and cell counts from ten replicate counting chamber wells.

The impact of Cu(II) exposure, inferred from ammonia oxidation associated specific oxygen uptake rates (sOUR) in both exponential and stationary phase cultures was statistically not dissimilar for Cu(II) doses lower than 1000 µM Cu(II), as inferred from a deviation from a value of unity (Figure 2). At 1000 µM Cu(II), exponential phase cultures were significantly more inhibited, (α = 0.05, Figure 2). The computed non-competitive inhibition coefficient based on intracellular Cu concentrations for sOUR was 5.6±0.6 fg Cu/µm^3^ cellular volume for exponential cultures (expressed as average ± standard deviation computed for best-fit parameter estimates as described in [13], [14]). Computation of similar K_I_ estimates for stationary phase cultures was precluded by the non-systematic trends in sOUR in response to Cu(II) doses (Figure 2).

**Figure 3:**
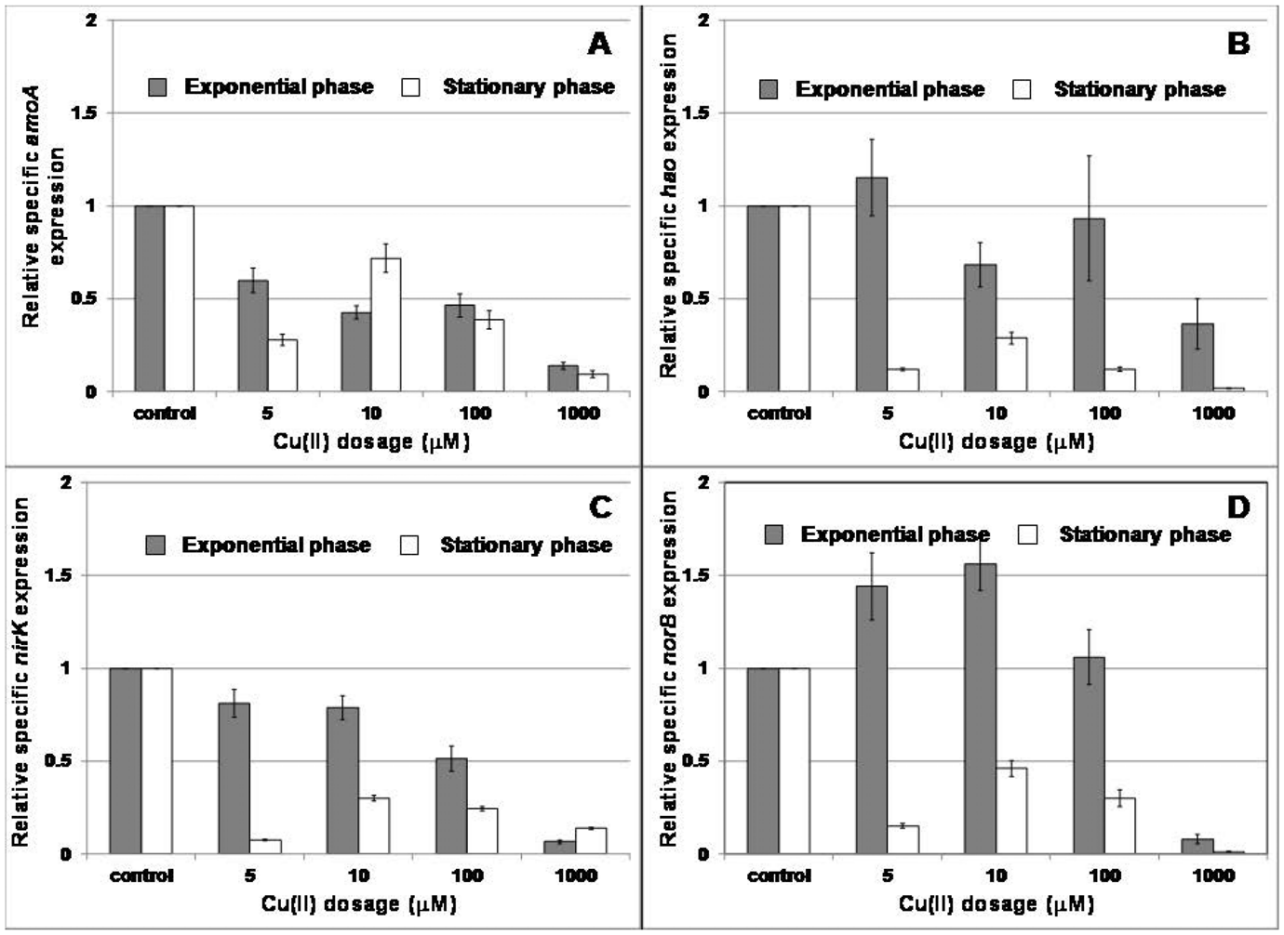
Impact of Cu(II) exposure on gene expression in stationary and exponential phase *N. europaea* cultures.

In general, there was a significant decline in the expression of *amoA* in both exponential phase and stationary phase cultures at all Cu(II) doses (Figure 3A). In contrast, the reduction in expression of *nirK* and *norB* was more severe for stationary phase cultures than for exponential phase cultures (Figure 3B–D). A statistically significant stimulation of *norB* expression (α = 0.05) was observed in exponential phase cultures at Cu(II) doses of 5, 10 and 100 µM (Figure 3D). Stimulation of *hao* expression was observed only at a Cu(II) dose of 5 µM for exponential phase cultures alone (Figure 3D).

Based on previous studies, stimulation of whole cell activity by Cu(II) in *N. europaea* has not been observed [15], which is in keeping with the results herein, although at doses of 5 µM and 10 µM, the level of inhibition was also not statistically significant (Figure 2). Stimulation by Cu(II) in *N. europaea* appears to be restricted to cell-free extracts for Cu(II) doses in the range 0–1000 µM [15], and expression of select genes (based on this study). The previously observed lack of stimulation of whole cell activity might be due to the absence of induction of *amoA* at sub-inhibitory Cu(II) doses. Alternately, it could be possible that AMO possesses a high affinity for Cu given that it catalyzes one of the most important reactions in the energy metabolism of *N. europaea* and is therefore not stimulated at low Cu(II) doses. It should be noted that lack of *amoA* induction is in contrast to the Cu(II) induced transcriptional induction of particulate methane monooxygenase (pMMO) expression [16], which is evolutionarily related to AMO [17].

On the other hand, stimulation of *norB* and to an extent *hao* expression by Cu(II) doses in exponentially growing cells cannot be conclusively explained, given that Nor and HAO both contain Fe co-factors. It could be speculated that the observed stimulation is a result of substitution of Fe(II) by Cu(II) in these two enzymes. Although not experimentally confirmed for *N. europaea* in this study, substitution of Fe(II) by Cu(II) has indeed been shown for phytoplankton as a means to overcome Fe limitation [18]. In contrast, the significant decline in the expression of *hao*, *nirK* and *norB* in Cu(II) exposed stationary phase cells suggests that under starvation and the associated lower energy fluxes during stationary phase, there might not be enough incentive for the cells to modulate expression of catabolic pathways, especially when faced with Cu(II) shocks.

Notwithstanding the impact of Cu(II) on the mRNA concentrations of *amoA*, *hao*, *nirK* and *norB*, it should be noted that mRNA concentrations may not capture the true complexity of the responses at the enzyme concentration or enzyme activity level. At the very least, it has been shown that there is a difference in the relative rapidity of responses at the mRNA and whole cell activity for these four genes [19], [20]. However, changes at the mRNA reflect one of the most fundamental responses of a cell to changes in its environment and could be precursors for more deliberate whole cell impacts [10], [21], [22], [23].

**Figure 4:**
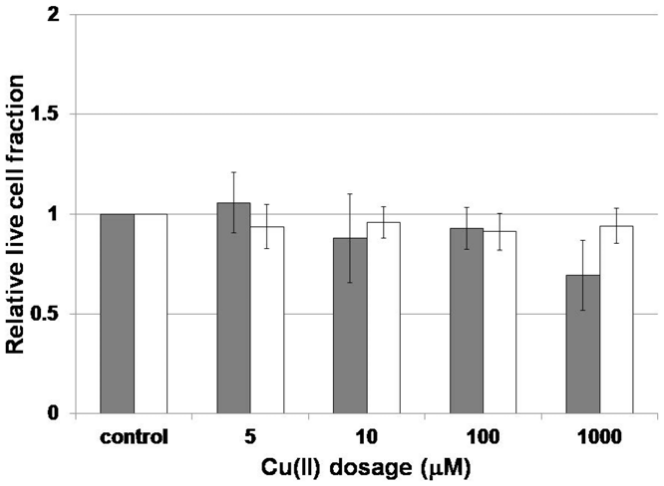
Impact of Cu(II) exposure on membrane integrity in stationary and exponential phase *N. europaea* cultures. Error bars depict cell counts from ten replicate fluorescence images.

Membrane integrity was not statistically impacted for both exponential phase and stationary phase cultures for Cu(II) doses lower than 1000 µM., At 1000 µM, however, exponential phase cultures but not stationary phase cultures displayed a statistically significant decrease (α = 0.05) in the fraction of cells with intact membranes (Figure 4).

The inhibitory impact of Cu(II) on nitrifying and non-nitrifying bacteria has been widely measured, in terms of specific ammonia and hydroxylamine oxidation rates, expression of *amoA* and select proteins [24], [25], [26], membrane integrity [10], mixed microbial community composition [27], global transcriptome profiles [28] and propensity for biofilm formation [29]. However, one limitation of these past studies has been the lack of directly measured intracellular Cu concentrations, which in principle should govern the cellular response, but have been traditionally difficult to measure [4]. Thus, one of the highlights of this work has been to link physiological state, uptake of a toxic metal and measured toxicity responses with experimentally measured intracellular Cu concentrations.

### Impact of physiological growth state on elemental composition and enrichment factors

In uninhibited control cultures, the intracellular concentrations of all elements except Zn were statistically not dissimilar during exponential growth and stationary phases at the confidence level α = 0.05 (Tables 1 and 2). The concentrations of P and S were consistently higher (α = 0.05) than the other elements in both growth phases (Tables 1 and 2). The higher intracellular P and S concentrations compared to other elements in *N. europaea* (Table 1) could be linked to the presence of these two elements in a plethora of intracellular molecules. These molecules include nucleic acids, phospholipids, energetic compounds rich in phosphodiester functional groups (for phosphorous) and amino acids (methionine, and cysteine), vitamins (thiamine, biotin and lipoic acid), coenzyme A, proteins with sulfhydryl moieties, and mediators of oxidative stress including glutathione (for sulfur) [1].

A high degree of intracellular elemental enrichment relative to the external bulk medium was also observed for inhibited cultures. In the control cultures harvested during exponential phase, the average enrichment factors of P, S, Fe and Zn were 190±63, 46±13, 14000±4700 and 2600±770, respectively (average ± standard deviation). In stationary phase control cultures, the average enrichment factors of P, S, Fe and Zn were 210±24, 52±6.0, 14000±1300 and 1600±300, respectively (average ± standard deviation). Intracellular Cu concentrations were statistically not dissimilar in the exponential growth and stationary phase cultures only for the control (Tables 1 and 2). The corresponding Cu enrichment factors for the exponential cultures were 2300±660, 670±170, 510±93, 880±210 and 150 (done without replication) for the control, 5 µM, 10 µM, 100 µM and 1000 µM, respectively (average ± standard deviation). For stationary phase cultures, the corresponding enrichment factors inside the cell were 1900±350, 340±51, 340±80, 340±320 and 48±9.0 for the control, 5 µM, 10 µM, 100 µM and 1000 µM, respectively(average ± standard deviation).

### Comparison of elemental profiles in *N. europaea* and *P. fluorescens*

**Figure 5:**
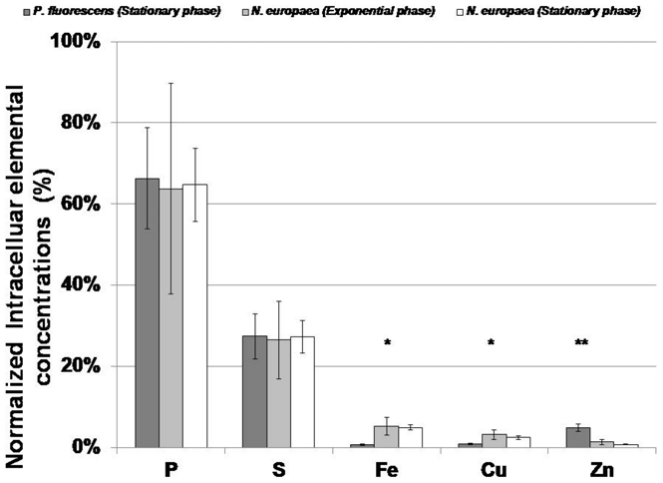
Comparison of elemental profiles in exponential and stationary phase cultures of *N. europaea* with stationary phase cultures of *P. fluorescens* [**5**]. Error bars depict standard deviation of 5–9 replicates in this study and 5 replicates in [5]. * represents the elements for which the molar fractions in *N. europaea* were statistically higher (α = 0.05). ** represents the elements for which the molar fractions in *N. europaea* were statistically lower (α = 0.05). The molar fractions of remaining elements were statistically not dissimilar (α = 0.05).

The elemental profiles of stationary phase cultures of *N. europaea* and *P. fluorescens* (obtained from a previous study [5]) were rather distinct. The comparison was restricted to stationary phase cultures since this was the focus of the previous study [5]. To facilitate the comparison, the intracellular molar concentrations of different elements were normalized to sum of the intracellular molar concentrations of P, S, Fe, Cu and Zn and expressed as a percentage. Based on the comparison, the molar fractions of P and S were statistically not dissimilar in stationary phase cells of *N. europaea* and *P. fluorescens* (α = 0.05, Figure 5). The molar fractions of Zn were statistically lower in *N. europaea* relative to *P. fluorescens* (α = 0.05, Figure 5). In contrast, the molar fractions of Fe and Cu were statistically higher in *N. europaea* (α = 0.05, Figure 5). The higher intracellular molar fractions of Fe and Cu were in keeping with our second hypothesis and are probably related to the high content of cytochromes and Cu containing enzymes in *N. europaea*. - which has been speculated [9], [15], but never before analytically demonstrated *in-situ*.

The tendency of *N. europaea* cells to accumulate high proportions of intracellular Cu and Fe relative to other elements underscores the high premium placed by *N. europaea* cells to take up and sequester these elements. Cu and Fe are thought to be integral parts of several components of the predominantly chemolithoautotrophic pathways of energy metabolism in *N. europaea* (as suggested by [9]) which could explain the experimentally measured relative enrichment of *N. europaea* cells in Cu and Fe compared to *P. fluorescens* (Figure 5, discussed below). The active role of Cu in AMO activity has been speculated as well [30]. Cu and Fe are also present in a cluster of enzymes that are related to the transfer of electrons to either oxygen or nitrite [9]. Of these Cu is present in a “blue” copper oxidase protein that is only aerobically expressed [31] as well as in a putative nitrite reductase enzyme (NirK) [9]. The other two enzymes in this protein cluster are heme group containing cytochromes [9]. *N. europaea* also possesses a diverse array of mechanisms to sequester and harvest Fe from the environment, presumably to give it a competitive edge in Fe limited settings. Approximately 14% of the genes coding for transporters (or 40 open reading frames) in its genome are directed towards Fe transport [9]. Additionally, although *N. europaea* does not synthesize siderophores other than citrate, the relatively high Fe content of *N. europaea* in environmental microbial assemblages is potentially sustained by virtue of its taking up siderophores synthesized by other organisms [9].

In sum, elemental profiling of *N. europaea* provided explicit evidence in support of previous speculation *and* genomic evidence related to the high selective uptake of Cu and Fe in *N. europaea* as well as possible insights into the physiological and toxicity responses. However, a direct comparison of XFM with cell-lysis based methods for elemental analysis was precluded by the absence of such documented data for *N. europaea*. As more environmentally or clinically important and relevant bacteria are interrogated at the elemental level, more insights can be gained into how intracellular elemental concentrations are impacted by and in-turn govern the interactions between cells and the environments to which they are exposed.

## Materials and Methods

### Cell cultivation

*N. europaea* (ATCC 19718) was grown in dark in batch reactors (V = 4L) at T = 21°C in a medium containing 280 mg/L ammonia-nitrogen and, in addition, (per liter): 0.2 g of MgSO_4_•7H_2_O, 0.02 g of CaCl_2_•2H_2_O, 0.087 g of K_2_HPO_4_, 2.52 g EPPS (3-[4-(2-Hydroxyethyl)-1-piperazine] propanesulfonic acid ) buffer, 1 mL of 13% EDTA-Fe^3+^, 1 mL of trace elements (10 mg of Na_2_MoO_4_•2H_2_O, 172 mg of MnCl_2_•4H_2_O, 10 mg of ZnSO_4_•7H_2_O, 0.4 mg of CoCl_2_•6H_2_O, and 100 mL of distilled water), 0.5 mL of 0.5% phenol red, and 0.5 mL of 2 mM CuSO_4_•5H_2_O. The reactor was mechanically mixed with magnetic stirring at 200 rpm and aerated with filter sterilized (0.2 µm) laboratory air provided at 3 L/min. Reactor dissolved oxygen (DO) concentrations were not monitored online, but varied in the non-limiting range 5–8 mg O_2_/L, based on discrete measurements performed using a Clark-type electrode (Yellow Springs Inc., Yellow Springs, OH). Cultures were monitored by measuring ammonia (potentiometry [32]), nitrite (colorimetry [32]) and cell concentrationa (from ten replicate wells in a cell counting chamber, Hawksley Scientific, U.K.). Reactor pH was controlled at 7.5±0.1 using automated addition of a 50 g/L sodium bicarbonate solution. Membrane integrity (BacLight™, Invitrogen, Carlsbad, CA) and ammonia oxidation associated specific oxygen uptake rates (sOUR, [10]) were measured in Cu(II) exposure studies. Samples for soluble and total Cu(II) analysis via atomic absorption spectrometry (Buck Scientific, Model 200A, East Norwalk, CT) were acidified to a pH of approximately 2.6 with 1N HNO_3_ and stored at 4°C.

### Cu(II) exposure studies and K_I_ estimation

Inhibition studies were conducted by exposing substrate-sufficient exponential batch and substrate-starved stationary batch *N. europaea* cultures to four discrete Cu(II) concentrations (5, 10, 100 and 1000 µM) for 4 h over and above the medium Cu(II) concentration of 1 µM. The impact of Cu(II) exposure was measured in terms of changes in cell concentration, membrane integrity, sOUR and intracellular concentrations of several elements. Inhibition of sOUR upon Cu(II) exposure was described using a non-competitive inhibition model as described previously [10], by correlating directly measured intracellular Cu(II) concentrations with reduction in cellular oxygen uptake rates.

### Functional gene expression

Expression of *amoA*, *hao*, *nirK* and *norB* was quantified and normalized to expression of the 16S rRNA gene using recently developed quantitative reverse transcriptase polymerase chain reaction (q-RT-PCR) assays [20]. Cells were lysed using Trizol® (Invitrogen, Carlsbad, CA) followed by total RNA isolation via sequential phase separation, RNA precipitation, and washing. Reverse transcription and DNA removal was performed using the QuantiTect® Reverse Transcriptase kit (Qiagen, Valencia, CA). Quantitative PCR (qPCR) was conducted in duplicate on an iCycler iQ™5 Multicolor Real-Time PCR Detection System (Bio-Rad Laboratories, Hercules, CA) using SYBR Green chemistry. Six point standard curves for qPCR were generated using decimal dilutions of pCR®4-TOPO® plasmids (TOPO TA Cloning® for Sequencing, Invitrogen) containing cloned PCR product inserts.

### Cell fixation and synchrotron XFM analysis

Cells were processed for XFM analysis using a standard protocol followed at the Advanced Photon Source (APS) at the Argonne National Laboratories (ANL), as previously described [5]. Briefly, cells were fixed in 2.5% glutaraldehyde for 1 h at 4°C, washed with sterile Millipore® water (≥18.3 MΩ), deposited on 200-mesh Formvar-coated transmission electron microscopy Au grids, and air-dried in a dust-free environment. The position of the cells on the grid was recorded visually by phase contrast microscopy for subsequent positioning of the synchrotron beam. *N. europaea* cells were distinguished from chemical precipitates based on their distinct morphology, determined extensively from previous studies [10], [19], [20]. Fixed cells were analyzed and imaged on the synchrotron x-ray microbeam at beamline 2-ID-D at APS-ANL, as described previously [5]. Quantitative processing of synchrotron XFM scans was conducted using MAPS software [33] (Figure 1). Cellular volume was calculated by multiplying the cell cross-section area by an average cell thickness of 1 µm obtained from the synchrotron XFM scans (data not shown). Intracellular elemental concentrations were expressed as mass per unit cell volume (femto-g/µm^3^ cell volume) or as parts per million (ppm), assuming a cell specific gravity of 1 g/ml, to facilitate comparison with a previous study [5]. On average 5–9 cells were subjected to XFM based elemental analysis, which is comparable to that published previously [5]. Concentrations of elements related to freely diffusible ions such as Cl^−^, K^+^ and Ca^2+^ could not be determined unequivocally and therefore were not considered for further interpretation.

### Nomenclature

Unless explicitly stated, elements are represented by their respective symbols without assuming any specific oxidation state. For instance, Cu(II) refers to divalent copper and Cu refers to copper in general or total copper concentrations.
